# The role of dog therapy in clinical recovery and improving quality of life: a randomized, controlled trial

**DOI:** 10.1186/s12906-024-04538-7

**Published:** 2024-06-12

**Authors:** Veronika Mittly, Veronika Fáy, Natália Dankovics, Vanda Pál, György Purebl

**Affiliations:** 1 Institute of Behavioral Sciences, Semmelweis University, Nagyvárad square 4, Budapest, 1089 Hungary; 2 South-Pest Central Hospital National Institute of Haematology and Infectology, Center for Rehabilitation, Jahn Ferenc street 62-66, Budapest, 1195 Hungary

**Keywords:** Animal assisted intervention, Dog therapy, Quality of life, Rehabilitation, Hungary

## Abstract

**Background:**

Any illness places a significant burden on patients, including deterioration in quality of life. Animal assisted therapy may be helpful in the rehabilitation process and in the treatment of patients to alleviate this phenomenon.

**Methods:**

A randomized, controlled research was conducted in 2019 and 2020 in a rehabilitation center in Budapest. In our study, the control and experimental groups received the same therapy, but the rehabilitation treatment of the intervention group was complemented by dog therapy. Patients were evaluated by means of the short form Beck Depression Inventory, State-Trait Anxiety Inventory, Illness Intrusiveness Ratings Scale, Visual Analog Scale for pain and the WHO-5 Well-being Index. For statistical analysis paired T-test and ANCOVA was performed.

**Results:**

58 participants in both groups took part in the research. Results confirm that both groups showed statistically significant improvement in all outcome measures, except for depression symptoms in dog therapy group. Quality of life of the participants improved significantly, their pain and anxiety levels were significantly reduced, they felt significantly less burdened by the disease in their daily lives. Therapeutic-dog sessions had a large effect on patients’ quality of life and anxiety.

**Conclusions:**

There is a strong case for animal assisted therapy as a complementary therapy in the rehabilitation program, and it is proposed that consideration should be given to the application of this method on a larger scale within health care. The study was retrospectively registered at ISRCTN Registry (registration number: ISRCTN10208787) on 15/03/2022.

## Background

The use of animals for therapeutic purposes goes back decades [1, 2]. Since the 1960s, animal-assisted interventions have become a widely used therapeutic method for children, adults and elderly patients. Several studies have demonstrated the health benefits of human-animal bonding [3–5]. Today, therapy animals are used effectively both in inpatient and outpatient settings [2]. The presence of a therapy animal is seen by clinicians as an adjuvant therapeutic option, which can greatly assist clinicians or therapists in the biopsychosocial approach to patient management [6–8]. Animal-assisted interventions are development activities that involve working with therapy animals to achieve specific goals (education, treatment, etc.) [2, 3]. In practice, animal-assisted intervention (AAI) takes the form of animal-assisted activity (AAA) or animal-assisted therapy (AAT). All forms of animal-assisted intervention constitute a promising adjunctive treatment that requires further research to scientifically demonstrate its efficacy and cost-effectiveness.

The effects of animal-assisted therapy in neurorehabilitation have also been investigated in a number of studies. A Canadian research team investigated the effects of dog therapy on stroke patients. At the end of the dog therapy participants showed improvement in their gait pattern and were able to move faster with the dog in comparison to walking with a cane [9]. A US study examined patients with nonfluent aphasia who had suffered a left hemisphere cerebral infarction years earlier. Patient satisfaction questionnaire data were assessed and found that during the 12 weeks of speech therapy with a therapy dog, patients became more motivated and willing to participate in sessions, were more comfortable talking about their emotional states, and also had an increase in spontaneous verbalisation compared to conventional speech therapy [10]. The benefits of animal-assisted therapy have also been reported in people with Parkinson’s disease, with dog therapy demonstrating a positive effect on motor performance, mood and sleep [11]. Several experts recommend the introduction of canine-assisted therapy for these patients to improve gait and balance, to treat depression, mood disorders, apathy and anxiety often associated with the disease [12]. In a multicentre randomised controlled trial among patients with multiple sclerosis, dog therapy significantly improved the quality of life in the intervention group improving balance and coordination [13]. In Hungary, a study was conducted in 2014 among patients treated at the Department of Spinal Cord Injuries of the National Medical Rehabilitation Institute. Their observations showed that dog therapy reduced the anxiety of patients, made them more open and eager to establish contact with each other and the therapy dog, and finally, the participants positively evaluated the effects of the therapy, as well [14]. A US case study presented the example of a 34-year-old woman who underwent a L4-S1 laminectomy and fusion for a discus hernia with significant complaints. The patient’s independence score and gait indicators at discharge all showed significant improvement compared to admission, and the female patient described the assistance of the therapy dog as a great motivational force [15]. 

In the field of musculoskeletal rehabilitation, animal-assisted therapy has also been proved to be beneficial in patients undergoing orthopedic surgery. In a randomized controlled trial Harper and colleagues studied 72 patients undergoing hip or knee replacement. After each canine therapy session, the patients indicated their pain levels on a visual analogue scale that were consistently significantly lower than within the control group that solely received physical therapy [1]. A group of US researchers also investigated the effect of animal therapy as a natural pain reliever. In a pain clinic for adult patients, dog therapy was used while patients were waiting for a doctor’s appointment. Their results showed that the patients who received dog therapy before the visit had significantly reduced pain and anxiety and were calmer and more relaxed [16]. 

To date, however, there is a paucity of randomized controlled multicenter studies with appropriate methodology, and the comparability of results is hampered by inconsistent terminology and methodology [2, 17]. The aim of our research is to demonstrate the potential place and relevance of dog assisted therapy as an adjuvant treatment in rehabilitation medicine. Furthermore, our aim is to demonstrate that therapy with dogs reduces patients’ anxiety and pain, improves their quality of life, and advances their complex rehabilitation. Our aim is to confirm the prominent, yet poorly researched and validated role of animal-assisted therapy in adult neuromusculoskeletal rehabilitation.

## Methods

Our research is a prospective, intervention-based, randomized controlled trial conducted in Hungary with balanced randomization [1:1]. Around 230.000 people live in Hungary with disabilities according to the Hungarian census in 2011. In our rehabilitation center, we treat around 1200 patients per year and approximately 150 patients at any one time. Since October 2019 we have introduced group dog therapy sessions once a week at our research center with a trained therapy dog and therapy dog handler. All group had a maximum of 5 participants, with 2 groups participating per week, for a period 3 weeks for each group. The dog was a 5-year-old wire-haired Hungarian Vizsla.

Patients in the study and in the control-group were selected both by stratified randomization, function- and symptom-oriented and transdiagnostic sampling. The subjects of our study were patients in our rehabilitation center, in Hungary. The study involved inpatients with neurological or musculoskeletal disorders. Patients with mild and moderate functional impairment were divided into control group and experimental group by a random number generator. Exclusion criteria were dog hair allergy, immunosuppressed status, early postoperative status, age less than 18 years, dementia, refusal to participate in research, and decompensated physical and psychological status determined by the treating physician. This study was performed in line with the principles of the Declaration of Helsinki. Approval was granted by the Ethics Committee of the Semmelweis University in 2019. Considering the fact that in Hungary the use of therapeutic animals is legally defined and may be performed only in accordance with a strict legal framework, the approval of an animal research ethics committee was not necessary. The welfare of the therapy dog was the responsibility of the therapy dog handler, the appropriate material and personnel conditions were provided by the “Kutyával az Emberért” Foundation, and our rehabilitation center. All applicable international, national guidelines for the care and use of animals were followed. The participants in our research provided informed written consent.

After informed consent, a questionnaire pack was completed with the subjects. The questionnaires used were validated measurement instruments in Hungarian. When compiling the questionnaire set, care was taken to ensure that the number of questions did not exceed the tolerable limit; therefore, shortened but valid versions were used wherever possible. We used a self-administered questionnaire on sociodemographic data such as age, residence, marital status, highest level of education, lifestyle factors such as activity, coping strategies and disease-specific information such as diagnosis, time since diagnosis, co-morbidities. In addition, the Beck Depression Inventory (BDI-9) to measure depression, the Spielberger State Anxiety Inventory (STAI) to define the rate of anxiety, the WHO Well-Being Index (WBI) to determine quality of life, the Illness Intrusiveness Rating Scale (IIRS) to rate general condition and the impact of the therapy received on different aspects of life, as well as the Visual Analogue Scale (VAS) to measure pain were all applied.

When completing the BDI-9 questionnaire, participants were asked to rate 9 statements from 1 to 4 points, according to how typical the statement was for them in the past 1 month (from 1-not typical at all, to 4-very typical) [18]. When completing the STAI questionnaire, participants were also asked to rate from 1 to 4 points how typical the statement was in view of their daily life (1-not at all, 4-absolutely typical) [19]. When completing the WBI, participants were asked to indicate the degree of activity, cheerfulness or calmness, they had experienced in the previous 2 weeks (0-not at all typical, 3-absolutely typical) [20]. In completing the IIRS, participants rated on a 7-point scale the extent to which their illness and/or its treatment affected different aspects of their lives, such as their activity, social relationships and work (1-not very much, 7-to a great extent) [21]. In addition, the VAS established the level of pain, with participants rating from 1 to 10, how severe their pain was at the time of completion (1-none, 10-the most severe ever) [22]. Psychometric properties of all questionnaires we use are well researched. Several studies confirm that the above mentioned questionnaires are valid and reliable screening instruments for use in healthcare patients [18, 20, 23–26]. 

Having completed the questionnaires, subjects who wished to participate in the study were randomized by a random number generator to an intervention or active control group. On weekdays members of both groups participated in a standard rehabilitation program, including physiotherapy, occupational therapy, electrotherapy and therapeutic massage, while on weekends participants did not receive therapy. Patients in both the intervention group and the control group received the same overall amount of therapy sessions, with an average of 2–3 h per day over a 3-week period. The minimum length of stay at our institution is 3 weeks, which is why we have set the duration of the intervention at 3 weeks. Our concept was to replace one physical therapy session in the animal-assisted intervention group with a dog therapy session, where patients received the same training exercise, but with the assistance of a therapy dog. The physiotherapy sessions, which were integrated into the rehabilitation program, also included complex functional development, typically including cognitive therapy as part of the motor rehabilitation therapy. During the intervention group therapy, by way of reflecting the group composition, we worked with the therapy dog handler to set up tasks designed to improve motor coordination and endurance, enhance fine motor skills of the hands, mobilize joints, improve balance, develop memory, reduce attention deficit, create peer support, reduce symptoms of anxiety and depression, and build and utilize group cohesiveness.

The emergence of the Sars CoV2 Coronavirus infection in Hungary in March 2020 and the resulting pandemic has fundamentally changed the way the healthcare system operates. The situation forced our research team to modify our research plan, due to a strict no-visit policy imposed on our rehabilitation center, which meant that the therapy dog handler and her dog were not allowed to enter the premises of our institution. Likewise, the organisation of group therapy sessions at the center was prohibited. In view of this uncertain situation, our research project was suspended. Consequently the inclusion of psychiatric patients was unrealised.

Data were processed using the IBM SPSS Statistics 26 statistical software, which was run in April 2021. In addition to descriptive analysis of the data, a paired T-test was applied to compare the mean pretest and posttest values of the two research groups and repeated measures ANCOVA was performed to establish whether there was a significant difference between the intervention group and the control group in terms of the scores on the questionnaires administered between the start and finish of the 3-week program. All tests were run with an alpha level of 0.05 (5%) and with a confidence level of 95%. The effect size of the conventional rehabilitation program and that of the dog therapy was expressed in eta squared (η2) and Cohen’s d.

## Results

**Fig. 1:**
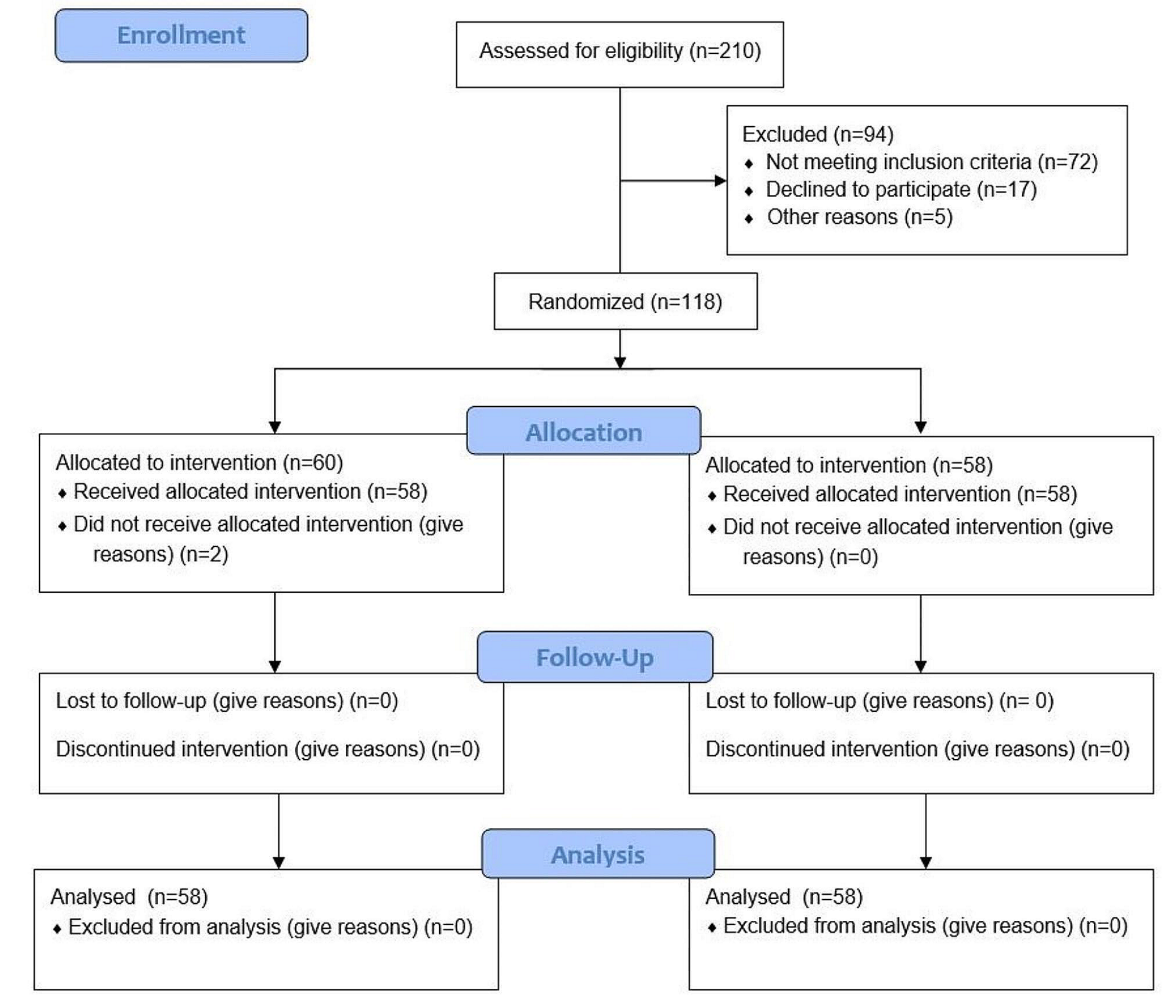
Flow chart of the study

As shown in the flow chart of our study, from October 2019 till March 2020, 118 patients in total participated in the research. (Fig. 1) Given that these individuals were studied over a 3-week period only, after randomization only 2 patients in total in the intervention group were unable to participate in the therapy sessions because their health deteriorated before the first session took place, resulting in their transfer from our center to another department.

In the intervention group (*N* = 58), the gender distribution of participants was dominated by women, as there was a predominance of women in the control group (*N* = 58). The age of the participants in both groups ranged from 18 years to patients in their 80s, with the dog therapy group being predominantly represented by patients aged 60–70 years, while the control group consisted of patients aged between 70 and 80 years. In both groups, the most common diagnoses for rehabilitation were degenerative arthrosis and cerebrovascular disease; polytrauma, lower limb amputation, joint endoprosthesis and herniated disc surgery were also evident. In both groups, the majority were in a relationship. Most of the participants in both groups lived in the capital and had a higher level of education. (Table 1) Patients with moderate to mild functional impairment in terms of functional capacity were included in both the control and experimental group. According to the legislation in force at the time of the study, the therapy dog was not allowed to enter the wards or the department, therefore, immobile patients were unfortunately excluded from the study.

**Table 1:** Distribution of study participants by sex, age, diagnosis, matrial status, highest level of education and residence

		Control group	Experimental group
**Gender**	Female (n)	32	41
	Male (n)	26	17
**Age (years)**	18–30 (n)	2	3
	31–40 (n)	2	9
	41–50 (n)	3	8
	51–60 (n)	6	11
	61–70 (n)	16	20
	71–80 (n)	23	6
	Over 80 (n)	6	1
**Diagnosis**	Stroke (n)	7	13
	arthrosis, lumboischialgia (n)	20	15
	Joint prosthesis, operated discus hernia (n)	13	9
	mono-, multi- and polytrauma (n)	10	9
	Multiple sclerosis (n)	3	2
	Amputation (n)	7	10
**Matrial status**	Single (n)	25	26
	In a relationship (n)	33	32
**Highest level of education**	Secondary school (n)	7	4
	High school (n)	18	11
	Associate’s degree (n)	22	28
	University (n)	11	15
**Residence**	Budapest (n)	36	42
	Larger city (n)	1	0
	Other city (n)	18	15
	Village (n)	3	1

**Fig. 2:**
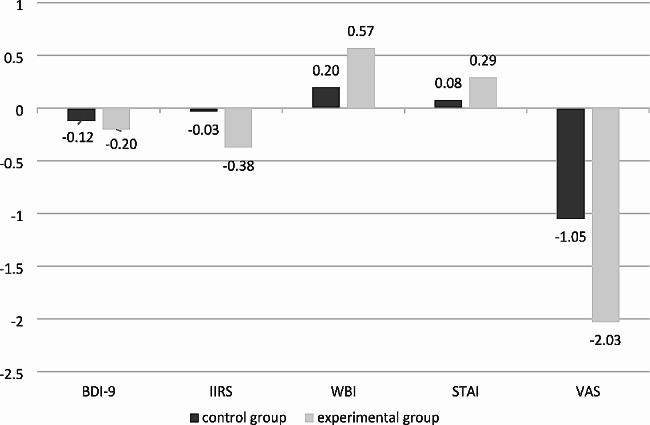
Changes between the initial and follow-up mean scores in each group

Regarding the *BDI-9* questionnaire measuring depression symptoms, the mean score for the statements decreased in both groups, but the changes showed a significant (*p* < 0.001) difference between the initial and follow-up scores of the participants only in the control group, based on the paired T-test results. ANCOVA test showed a significant (*p* < 0.001) difference between the effect of dog therapy (Cohen’s d = 0,344) and conventional rehabilitaion program (Cohen’s d = 0.797). (Fig. 2) (Tables 2 and Tables 3).

When assessing the *Illness Intrusiveness Rating Scale*, paired T-test showed a significant difference (*p* < 0.001) between the initial and final scores of both study groups, meaning that patients felt less affected by some aspects of their lives due to their illness as a result of both the rehabilitation program (Cohen’s d = 0.663) and the impact of dog therapy (Cohen’s d = 0.779). According to ANOVA test there was no significant difference between the two study groups. (Fig. 2) (Tables 2 and Tables 3).

**Table 2:** Outcomes at follow-up time-point within groups and between groups

	Control group						Between-group (repeated measures analysis of variance)		Experimental group					
	Baseline mean	Baseline SD	Follow up mean	Follow up SD	*P*	adj. *p*	*p*	Adj. *p*	Baseline mean	Baseline SD	Follow up mean	Follow up SD	*p*	Adj. *p*
BDI-9	1.786	0.561	1.665	0.498	< 0.001	< 0.001	< 0.001	< 0.001	1.502	0.398	1.299	0.337	0.063	0.314
IIRS	2.756	0.934	2.722	0.862	< 0.001	< 0.001	0.228	2.964	3.166	1.267	2.790	1.158	< 0.001	< 0.001
WBI	2.386	0.702	2.582	0.651	< 0.001	< 0.001	0.002	0.01	2.593	0.79	3.162	0.572	< 0.001	< 0.001
STAI	2.702	0.578	2.778	0.561	< 0.001	< 0.001	0.007	0.133	2.866	0.537	3.158	0.475	< 0.001	< 0.001
VAS	5.31	2.44	4.26	2.048	< 0.001	< 0.001	0.231	0.231	5.29	2.804	3.26	2.26	0.001	0.005

SD = standard deviation, p = level of significance, adj. p = Bonfferoni adjusted p value

**Table 3:** Effect sizes of conventional rehabilitation program and dog therapy

		F (1;114)	η2	Cohen’s d
BDI-9	Control group	54.519	0.324	0.797
	Experimental group	3.529	0.030	0.344
IIRS	Control group	32.872	0.224	0.663
	Experimental group	22.079	0.162	0.799
WBI	Control group	110.939	0.493	0.984
	Experimental group	22.256	0.187	0.857
STAI	Control group	88.271	0.436	0.926
	Experimental group	29.824	0.207	0.903
VAS	Control group	112.953	0.498	0.989
	Experimental group	11.454	0.091	0.599

F = effect of conventional rehabilitation program and effect of animal assisted therapy, η2 and Cohen’s d = effect size of conventional rehabilitation program and of animal assisted therapy

The results of the *WHO Well-Being Index*, which measured the quality of life of the participants, also showed that the patients’ quality of life indicators improved significantly during the research procedure in both groups based on paired T-test results (*p* < 0.001). Based on ANCOVA, a significant difference between the effect of the rehabilitation program (Cohen’s d = 0.984) and the dog therapy (Cohen’s d = 0.857) was seen. (*p* < 0.002) (Fig. 2) (Tables 3 and 2).

The *Spielberger State Anxiety Inventory* also found a statistically significant difference between participants’ initial and final self-perceptions for participants in both groups (*p* < 0.001). After Bonferroni correction, the ANCOVA test confirmed that there was no significant difference between the effect of the rehabilitation program (Cohen’s d = 0.926) and the animal assisted therapy (Cohen’s d = 0.903). (Fig. 2) (Tables 2 and Tables 3).

The *Visual Analogue Scale* was used in our study to assess participants’ pain. Paired T-test results showed a significant difference between the initial and follow-up scores of both experimental (*p* = 0.001) and control groups (*p* < 0.001). The mean pain rating decreased at the end of the program. Repeated measures ANCOVA showed that there was no significant difference in pain-relief between the control group (Cohen’s d = 0.989) and the dog therapy group (0.599) compared to each other. (Fig. 2) (Tables 2 and Tables 3).

## Discussion

In the study, our aim was to demonstrate that a specially trained therapy dog with the assistance of a specially trained therapy dog handler can be used as an adjuvant therapy option in everyday patient care, effectively complementing traditionally used treatment methods such as pharmacotherapy, physiotherapy, physical therapy, psychological support, cognitive therapy, speech therapy, sports therapy, occupational therapy, conductive pedagogy, and art therapy.

The results show that both the conventional rehabilitation program and the rehabilitation complemented with dog therapy significantly improved the quality of life of the participants whose pain and anxiety levels were significantly reduced. As a result, they felt significantly less burdened by the disease in their daily lives. Interestingly, the conventional rehabilitation program had a significant effect in reducing symptoms of depression, whereas the same was not true for dog therapy. Comparing the two study groups, significant differences were only found in the Beck Depression Scale and the WHO Well-being Index, in which the standard rehabilitation program has been shown to be more effective than dog therapy. Between two groups no difference was seen in the result of STAI, IIRS and VAS for pain, so dog therapy proved to be as effective as conventional rehabilitation program. Effect size analysis (Cohen’s d) showed that both the standard rehabilitation program and the dog therapy had a large effect on the participants’ quality of life and anxiety. In addition, although not significant, a greater effect was measured for dog therapy in terms of the impact on burden of disease compared to the standard rehabilitation program. In terms of pain reduction, a large effect was found in the control group, while the intervention group showed a medium effect. For the conventional rehabilitation program, a medium effect size was calculated for the effect on depression symptoms, while a small effect was seen for dog therapy.

These results are inconsistent with literature demonstrating the effectiveness of AAT in a range of mental disorders [27–30]. There are several possible explanations for these findings, all of which are important limitations of our study. Firstly, the current dog-assisted treatment programme was not designed to specifically target depressive symptoms. Second, the inability to create homogeneous patient groups also made it difficult to detect small but important changes. Third, the current sample size limited the ability to create subgroups of participants. Fourthly, it was not possible to investigate the specific active elements of the dog-assisted therapy within the current experimental design.

On the other hand, the limited efficacy of dog-assisted therapy for specific symptoms described above also raises the question of the added value of AAT over conventional therapies. It is important to emphasise that our results show that dog-assisted therapy can have a beneficial effect on several aspects of patients’ condition, such as physical, psychological and social well-being and quality of life. These beneficial effects were seen in patients with a wide range of neurological, rheumatological or orthopaedic-traumatological conditions, with an overall positive effect. It is also important to highlight the positive impact of dog-assisted therapy on patient engagement and motivation. The patients in the intervention group were always eager to participate in the dog therapy sessions and, in addition to completing the questionnaires, their individual experiences and comments also showed that the therapy animal had a positive impact on their daily lives. Our results also confirmed that dog therapy is an effective therapeutic option in the rehabilitation process as it promotes the development of patients and helps them to maintain and increase their motivation in the above mentioned therapies. There were no serious adverse events reported as being intervention-related. Since our research was limited especially by the outbreak of the coronavirus epidemic, we plan to continue the work with our original objectives in the future, and a possible long-term follow-up study is also under consideration. As the intervention was implemented for both sexes, all ages, all diagnoses, and at different levels of functional loss, the results indicate that with the exception of immobile patients, the entire range of patients in neuromuskuloskeletal rehabilitation would benefit from using dog assisted therapy. These effects were confirmed by a systematic review on the subject, which showed that the use of a therapy animal during rehabilitation increased patients’ motivation, concentration, cognitive load, as well as fostering a positive and hopeful attitude towards their recovery, verbalization and social relationships with therapists and fellow patients [31]. As confirmed by several other studies and meta-analyses, the results of our research show that canine-assisted therapy can reduce participants’ anxiety and pain, alleviate their depressive symptoms, increase their activity and enhance their mood [2, 3, 32]. In addition, the therapy animal acts as an excellent social catalyst, helping to build relationships between patients and to develop social coping. This creates a cohesive force between the participants in the dog therapy session which, in our experience, is maintained in later life, enabling them to cope effectively with everyday problems and changes in their life situation [33]. In summary, although the specific elements, mechanism of action and cost-benefit values of dog-assisted therapies require further research, we can conclude that dog-assisted therapy is clearly a positive addition to conventional therapies, with considerable potential for patient engagement, and is therefore worthy of further investigation.

Further research is also needed to include patients with more severe conditions and greater functional impairment, which was not feasible in this study due to the legal environment in force at the time of the research. The above mentioned beneficial effects may be due to several phenomena that need further research, such as the direct effects of animal-assisted therapy on improving self-esteem and life satisfaction, neurobiological and biochemical facilitation of the development and strengthening of social bonds, and immune system function [34, 35]. 

The definition of applied methods to measure the impact of animal-assisted interventions is awaiting standardization and scientific consensus in the field, thus increasing the reproducibility and reliability of the studies. On the basis of the subjective opinion and experience of research participants and those conducting the procedure, the beneficial effects of therepeutic animals are boundless. However, it is still a serious challenge to capture and formulate these effects by established, conventional scientific methodology [36]. Animal-assisted interventions should also be observed and analyzed from the perspective of the therapy animal, to see how it is affected by the intervention itself. Several studies have shown that during an intervention, the animal’s heart rate may rise; it may wag its tail, bark, and increase eye contact, among other forms of reaction [28, 35, 37, 38]. 

It should also be noted that although the terms used in the context of animal-assisted intervention are clearly defined, researchers do not use these definitions consistently. In most cases, the boundaries between animal-assisted activity and therapy are blurred, leading to further confusion in interpretation. In addition, it is a common misconception to publish research findings on animal-assisted interventions with assistance animals, such as guide dogs or seizure dogs [2, 39, 40]. 

In view of international literature on the subject, it is interesting to observe that although the number of publications is increasing year by year, unfortunately there is a paucity of good quality research on animal assisted intervention since in most cases it is not possible to draw far-reaching conclusions from the study results due to a lack of randomization or control groups. This is a challenge as well for future research in this field, so that the effects of animal-assisted intervention can be demonstrated beyond doubt and thus gain a well-deserved place in the field of medicine.

## Conclusions

In conclusion, results show that dog-assisted therapy in the rehabilitation process of patients with musculoskeletal disorders improves physical and mental well-being of the participants to same extent as the standard rehabilitation program. The experience and research results of the past decades confirm that all forms of animal-assisted intervention are a promising field of adjuvant medicine that requires further research to scientifically prove their efficacy and viability. It would be worthwhile to carry out more randomized, controlled, multicenter studies in this field, and there is also an urgent need to develop a uniform terminology and methodology in the field of health sciences.

## Data Availability

The datasets used and analysed during the current study are available from the corresponding author on reasonable request.

## Abbreviations

AAI: Animal-assisted intervention
AAA: Animal assisted activity
AAT: Animal assisted therapy
ANCOVA: Analysis of covariance
BDI-9: Beck Depression Inventory
BSCI-LM: Brief Stress and Coping Inventory’s Life Meaningfulness subscale
IIRS: Illness Intrusiveness Rating Scale
Sars CoV2: Severe acute respiratory syndrome coronavirus 2
SD: Standard deviation
STAI: Spielberger State Anxiety Inventory
VAS: Visual Analogue Scale
WBI: WHO Well-Being Index
